# A Combined Spectroscopic and Multivariate Analysis Approach for the Structural Characterization of Metal-Based Deep Eutectic Solvents: Choline Chloride and Cobalt Chloride Hexahydrate in Water Mixtures

**DOI:** 10.1021/acs.inorgchem.6c01344

**Published:** 2026-07-14

**Authors:** Giorgia Mannucci, Alessandro Tofoni, Matteo Busato, Mauro Giustini, Paola D’Angelo

**Affiliations:** Dipartimento di Chimica, 9311 Università degli Studi di Roma “La Sapienza”, P.le A. Moro 5, 00185 Rome, Italy

## Abstract

Ultraviolet–visible (UV–vis) spectroscopy, Co K-edge X-ray absorption spectroscopy (XAS), and multivariate curve resolution (MCR) were combined to investigate cobalt speciation in the choline chloride/CoCl_2_·6H_2_O (1:2) metal-based deep eutectic solvent and its aqueous mixtures (1:2:W, W = 0–50). UV–vis spectroscopy provided information on coordination geometry, while XAS yielded quantitative structural information on the short-range environment of the metal center. MCR analysis of the XAS data set resolved overlapping spectral contributions and enabled identification of distinct cobalt species. Three complexes were identified: tetrahedral [CoCl_4_]^2–^ (Co–Cl = 2.30(1) Å), mixed-ligand octahedral [CoCl­(H_2_O)_5_]^+^ (Co–Cl = 2.34(1) Å; Co–O = 2.09(1) Å), and fully hydrated [Co­(H_2_O)_6_]^2+^ (Co–O = 2.09(1) Å). Chloride-rich species dominate at low hydration, whereas increasing water content shifts the equilibrium toward hydrated octahedral complexes through progressive ligand substitution. These results provide a quantitative description of hydration-driven changes in cobalt coordination and demonstrate that the combined XAS–MCR approach is effective for resolving metal speciation in complex eutectic and multicomponent liquid systems.

## Graphical abstract

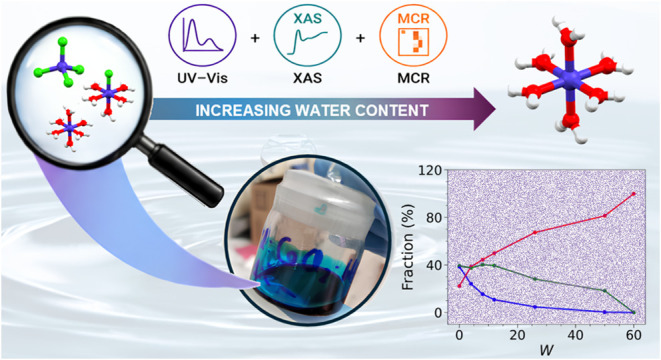

## Introduction

Deep eutectic solvents (DESs) represent a rapidly expanding class of green media that have attracted considerable interest owing to their distinctive physicochemical properties. Characterized by nonflammability, negligible vapor pressure, low toxicity, and high solvating ability, DESs offer environmentally friendly alternatives to conventional organic solvents.^1–3^ Although a universally accepted definition is still lacking, DESs are broadly described as mixtures of two or more compounds exhibiting a melting-point depression more pronounced than that predicted for the ideal eutectic composition.^4,5^

DESs are commonly categorized into five types, three of which incorporate a metal salt as one of their constituents.^3^ These metal-containing systems are collectively referred to as metal-based deep eutectic solvents (MDESs). MDESs combine the typical advantages of DESs such as straightforward preparation, no purification requirements, and adjustable physicochemical properties, with functionalities that enable diverse technological applications. Owing to their high ionic content, these systems exhibit significant polarity and conductivity, making them promising electrolytes and reaction media.^6–11^ Several MDESs have been successfully applied in electrodeposition processes, including systems based on choline chloride (ChCl) with chromium,^12,13^ copper,^14^ nickel,^15^ zinc,^16–19^ and cobalt salts,^20^ which allow the formation of high-quality metal and alloy coatings while offering alternatives to conventional, more hazardous baths. Beyond electrochemistry, mixtures containing transition-metal chlorides and hydrogen-bond acceptors have shown effectiveness in catalytic and extraction applications.^21–25^

Water plays a crucial role in defining the behavior of MDESs and can be present in the mixture in either desired or undesired ways. Indeed, many MDESs are markedly hygroscopic and readily absorb atmospheric moisture. Conversely, controlled water addition is widely employed to tune viscosity and polarity.^26–29^ Understanding how water influences the structural organization of MDESs is thus essential for rational solvent design and optimization for targeted applications.

A detailed structural characterization of systems containing metal species typically requires a combination of experimental techniques. Ultraviolet–visible (UV–vis) spectroscopy provides preliminary information on the electronic structure and coordination geometry of the metal ion,^30^ allowing qualitative distinctions among different coordination environments. However, intrinsic spectral broadening and overlap of electronic transitions limit its ability to yield unambiguous structural parameters. To overcome these limitations, X-ray absorption spectroscopy (XAS) is employed, as it enables a quantitative determination of local coordination numbers and interatomic distances around the absorbing metal center. When XAS measurements are performed on multicomponent systems in solution, the resulting spectra represent an average over all species present.^31^ In this context, multivariate curve resolution (MCR) analysis enables the decomposition of the experimental spectra into contributions from individual components, thereby overcoming this limitation and providing a more detailed description of the speciation in solution.^32^

In this work, we propose a combined spectroscopic strategy based on UV–vis absorption, XAS, and MCR to probe the structure of MDESs and their aqueous mixtures. The complementary sensitivity of these techniques enables the discrimination of electronic structure, coordination environment, and metal speciation in complex multicomponent systems, overcoming the intrinsic limitations of individual methods. As a case study, we investigate the ChCl/CoCl_2_·6H_2_O (1:2) MDES and its progressive dilution with water. This molar ratio was selected to resemble the composition of the archetypal DES reline (ChCl/urea 1:2), thereby facilitating comparison with previous studies on metal speciation in choline chloride-based eutectic systems.^33–37^ Cobalt is widely employed in lithium-ion batteries, advanced alloys with magnetic and mechanical functionality, and as a catalyst in key industrial processes.^21,38,39^ It is also relevant in electrodeposition; however, aqueous electrolytes are hindered by hydrogen evolution, which reduces efficiency and compromises deposit quality.^40,41^ In light of these considerations, cobalt-based MDESs offer a promising alternative medium for electrodeposition.^20^ Beyond the specific system examined, the integrated approach presented here is broadly applicable and can be extended to other MDES formulations, providing a general strategy to elucidate structure–composition relationships and hydration effects in complex metal-containing eutectic solvents.

## Experimental Section

### Chemicals and Sample Preparation

CoCl_2_·6H_2_O (≥99%) and ChCl (≥99%) were purchased from Merck and the former compund was used as received while the latter was dried under vacuum overnight at 60 °C. The components were mixed at the requested molar ratio in a glass test tube to obtain the ChCl/CoCl_2_·6H_2_O 1:2 MDES. Milli-Q water was added to the MDES to prepare ChCl/CoCl_2_·6H_2_O:water 1:2:W mixtures, with W = 4, 8, 12, 16, 26, and 50. The 50 mM aqueous CoCl_2_ solution was prepared by dissolving the proper salt amount in Milli-Q water.

### UV–Vis Spectroscopy

Absorption spectra of ChCl/CoCl_2_·6H_2_O:water mixtures at various 1:2:W molar ratios were recorded in the visible region at room temperature. Additionally, a 50 mM CoCl_2_ aqueous solution spectrum was collected as a reference. A Varian Cary 5E UV–vis spectrometer was used to carry out the measurements, with a quartz cell of 0.01 cm optical path length. Over the 250–1000 nm range, absorbances were recorded at intervals of 0.5 nm and with an integration time of 0.1 s. Because the samples were too viscous to measure their volumes accurately, the spectra were presented as the molal absorption coefficient of the Co^2+^ ion vs wavelength. The raw UV–Vis spectra were baseline-corrected using the SpectraGryph software with a linear background model. For all samples, the UV–Vis spectrum of pure water was used as the reference spectrum for baseline subtraction.^42^

### XAS Measurements

XAS spectra of ChCl/CoCl_2_·6H_2_O:water mixtures at different 1:2:W molar ratios were collected at the Co K-edge in transmission geometry at the Elettra-Sincrotrone Trieste (Italy) 11.1 beamline.^43^ An exact amount of each mixture was placed on a cellulose membrane, which was subsequently covered on both sides with Mylar tape due to the sample high metal concentration. A Si(111) double crystal monochromator was used for the measurements, and the storage ring was operating at 2 GeV with a 200 mA beam current. For every sample, a minimum of three spectra were collected and averaged. XAS data were also collected on a 50 mM CoCl_2_ aqueous solution as a comparative system.

### MCR Decomposition

A transformation matrix-based approach from the MCR family was applied to extract the spectral and concentration profiles of the principal components from the experimental XAS data set, using the PyFitit package.^44^ The optimal number of components was identified via a scree-plot statistical analysis. During the decomposition, one of the resolved spectral components was constrained to match the independently measured XAS spectrum of 50 mM CoCl_2_ in H_2_O.

### EXAFS Data Analysis

The analysis of the extended X-ray absorption fine structure (EXAFS) region of the XAS spectra was performed using the GNXAS code.^45,46^ Amplitudes and phase shifts were computed for clusters with fixed geometries, employing the muffin-tin (MT) approximation. The MT radii were selected to achieve approximately 20% overlap between adjacent MT spheres, with values of 1.60, 1.20, 0.90, and 0.20 Å assigned to Co, Cl, O, and H atoms, respectively. The Hedin-Lundqvist (HL) scheme’s advanced exchange-correlation self-energy models were applied to account for inelastic photoelectron losses in the final state.^47^ Theoretical signals derived from n-body distribution functions were calculated using the multiple-scattering (MS) theory and combined to reconstruct the overall theoretical contribution. Nonstructural parameters, such as the edge ionization energy (*E*_0_) and the energy positions and amplitudes of the KM_1_ and KM_2,3_ double-electron excitation channels, were also optimized. The pre-edge background was modeled by a linear function and subtracted from the raw absorption spectra.

### XANES Data Analysis

The analysis of the X-ray absorption near-edge structure (XANES) region of the XAS spectra was conducted using the MXAN code.^48^ The potential was calculated within the MT approximation, using a complex optical potential based on the local density approximation of the self-energy of the excited photoelectron.^49^ The real part of the self-energy was determined using the HL scheme,^50^ as the full complex HL potential is known to cause significant overdamping at low energies. As a result, inelastic losses were accounted for with a phenomenological approach by convoluting the theoretical spectrum with a Lorentzian function that has an energy-dependent width, Γ_tot_(*E*) = Γ_c_ + Γ_mfp_. Here, Γ_c_ refers to the core-hole lifetime, while the energy-dependent term Γ_mfp_ represents all intrinsic and extrinsic inelastic processes. Below an onset energy *E*_s_, Γ_mfp_ is zero, which in extended systems corresponds to the plasmon excitation energy. After this threshold, Γ_mfp_ increases from a value *A*_s_ following the universal form of the mean-free path in solids.^51^ The values of *E*_s_ and *A*_s_ are derived at each computation step (i.e., for each geometrical configuration) using a Monte Carlo fit. Experimental resolution is considered by further convoluting with an energy-independent Gaussian function. Least-squares fitting is performed by optimizing the geometry of the initial models through the minimization of a residual function *R*_sq_ defined as

1 $$R_{\mathrm{sq}}=\frac{\sum_{i=1}^{m}w_{i}{\left(y_{i}^{\mathrm{th}}-y_{i}^{\exp}\right)}^{2}}{\epsilon_{i}^{2}\sum_{i=1}^{m}w_{i}}$$

where *m* is the number of data points, *y_i_* ^th^ and *y_i_* ^exp^ are the theoretical and experimental values of the absorption, respectively, ϵ_*i*_ is the individual error in the experimental data set, and *w*_*i*_ is a statistical weight. During the minimization procedure five nonstructural parameters were optimized, namely the experimental resolution Γ_exp_, the Fermi energy level *E*_F_, the threshold energy *E*_0_, and the energy and amplitude of the plasmon *E*_s_ and *A*_s_.

## Results

### UV–Vis Spectroscopy

UV–Vis spectroscopy is highly sensitive to changes in the local environment of an absorbing center and therefore provides valuable qualitative insight into metal coordination. In this context, the Co^2+^ ion, owing to its partially filled d-orbitals, is particularly well suited for speciation analysis by UV–Vis methods, as variations in coordination geometry and ligand field are reflected in its visible absorption features.^52–56^

In this study, visible-region absorption spectra (Vis) were collected for ChCl/CoCl_2_·6H_2_O: water mixtures at different 1:2:W molar ratios (with W in the range 0 to 50) and compared with that of a 50 mM CoCl_2_ aqueous solution, allowing qualitative trends in the cobalt speciation upon hydration to be assessed (Figure 1). As shown in Figure 1, most of the investigated systems display spectral features indicative of the coexistence of two distinct coordination environments. In particular, an absorption band in the 400–600 nm region is observed, which is consistent with Co^2+^ ions in an octahedral ligand field,^52–56^ while a second band appearing between 600 and 750 nm suggests the presence of tetrahedrally coordinated cobalt species.^54,56,57^ The tetrahedral component exhibits a higher intensity, as expected from the larger molar extinction coefficients typically associated with tetrahedral Co^2+^ complexes. This behavior arises from the absence of an inversion center and the resulting partial p-orbital character, which relaxes the Laporte selection rules for d–d transitions.^58^

**Figure 1:**
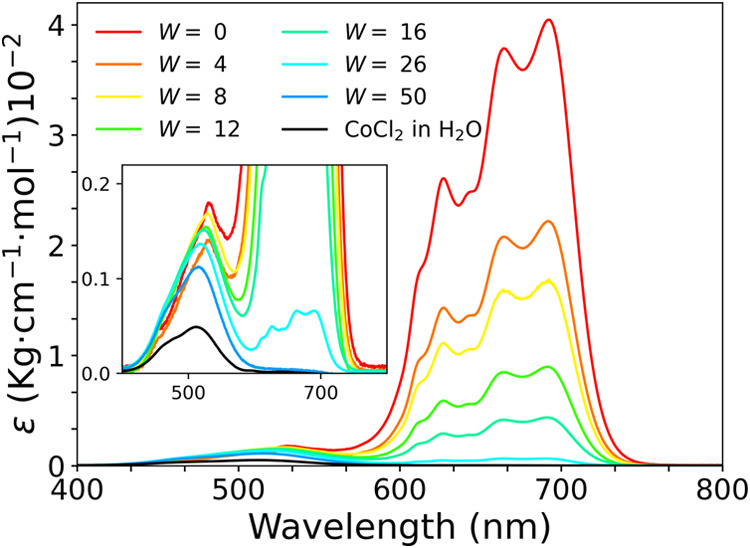
Vis absorption spectra of the ChCl/CoCl_2_·6H_2_O:water mixtures at different 1:2:W molar ratios and of a 50 mM CoCl_2_ aqueous solution (inset: magnification of the 450–750 nm spectral region).

The Vis spectrum of aqueous CoCl_2_ displays the typical signature of Co^2+^ in an octahedral ligand field (λ_max_ = 513 nm),^59,60^ with no absorption detected in the 600–750 nm region. This confirms that, in pure water, cobalt predominantly exists as fully hydrated octahedral complexes.^50,61–64^ Similarly, the 1:2:50 ChCl/CoCl_2_·6H_2_O:water mixture shows only the absorption band in the 400–600 nm range, indicating that at this dilution the Co^2+^ cation is mainly present as octahedrally coordinated species. However, the observed red shift of the absorption maximum as compared to the aqueous solution, together with the increase in molar absorptivity (ϵ_max_) suggests partial substitution within the first coordination sphere, consistent with the coexistence of water molecules and chloride ions around the metal center for some of the complexes, as previously reported.^61^ Note that chloride is a weaker-field ligand than water and its coordination reduces the crystal field splitting between ground and excited states. Moreover, although d–d transitions are formally dipole-forbidden in centrosymmetric octahedral environments, distortions from ideal symmetry can relax selection rules, resulting in enhanced absorption intensity.^61^

As the water content decreases, the absorption maximum of the band in the 400–600 nm region progressively shifts to longer wavelengths and ϵ_max_ increases. This trend indicates once more partial replacement of a coordinated water molecule by a chloride anion within the octahedral Co^2+^ first coordination sphere.

From the 1:2:26 ChCl/CoCl_2_·6H_2_O:water composition onward, an additional band emerges in the 600–750 nm region, characteristic of tetrahedral Co^2+^ species.^54,56,57^ The intensity of this band increases steadily as the water content decreases, while its position remains essentially unchanged. This behavior indicates that, in 1:2:W mixtures (W = 0–26), the same tetrahedral cobalt species is present throughout the composition range, with its relative abundance progressively diminishing upon increasing hydration.

While UV–Vis spectroscopy provides valuable qualitative insight into changes in the Co^2+^ coordination as a function of hydration, it does not allow coordination numbers and metal–ligand distances to be determined, nor does it enable a quantitative evaluation of the relative concentrations of coexisting species in the case of this metal center. These limitations motivate the use of complementary experimental techniques to achieve a more detailed and quantitative description of metal speciation in these complex systems.

### XAS Spectroscopy

When investigating metal ions in solution, XAS represents one of the most accurate techniques for determining the local structure around the absorbing atom.^50,64–68^ To obtain a quantitative description of the species present in the ChCl/CoCl_2_·6H_2_O:water mixtures, XAS measurements were performed at different 1:2:W molar ratios, together with a 50 mM aqueous CoCl_2_ solution used as a reference.

The normalized XAS spectra and the corresponding EXAFS signals are reported in Figure 2. Inspection of the XANES region reveals a clear and systematic evolution of both the white-line and pre-edge intensity as a function of water content. In particular, the white-line intensity increases, while the pre-edge intensity progressively decreases upon water addition. The pre-edge feature at approximately 7710 eV is associated with the dipole-forbidden 1s → 3d transition, whose intensity is highly sensitive to the local symmetry around the absorbing atom. Deviations from ideal octahedral coordination or the absence of an inversion center lead to partial relaxation of the selection rules through p–d orbital mixing, which is enhanced by reduced local symmetry.^69,70^ As shown in the inset of Figure 2a, the gradual decrease in the pre-edge intensity with increasing water molar ratio therefore indicates a progressive increase in local symmetry around the Co^2+^ ions, consistent with a transition toward a more regular octahedral coordination environment and the predominance of 6-fold hydrated Co^2+^ complexes.

**Figure 2:**
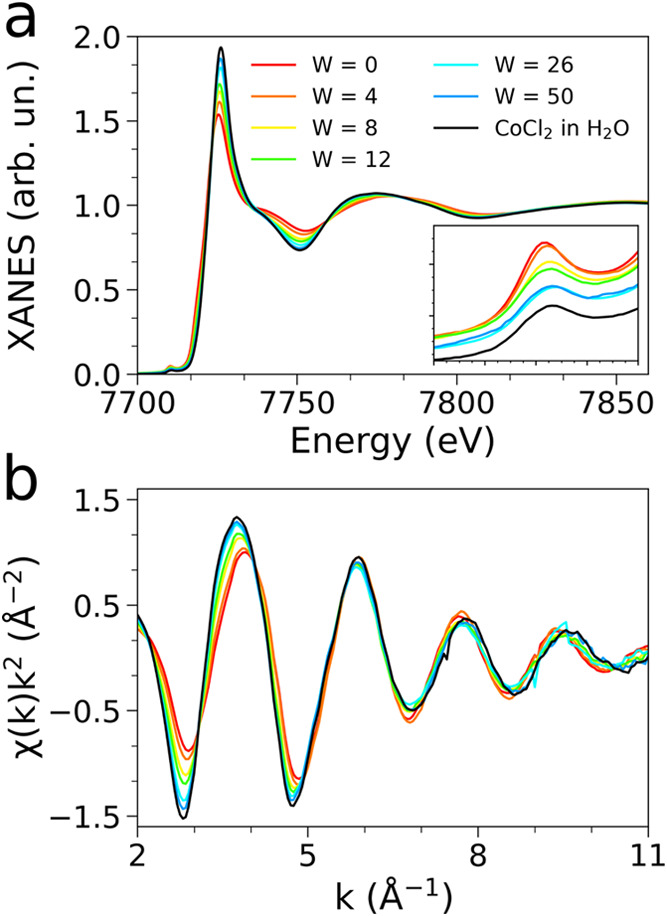
Co K-edge (a) normalized XAS and (b) EXAFS experimental spectra collected on ChCl/CoCl_2_·6H_2_O:water mixtures at different 1:2:W molar ratios and on a 50 mM CoCl_2_ aqueous solution. Inset: magnification of the pre-edge 1s → 3d transition.

In the EXAFS region (Figure 2b), which is highly sensitive to first-neighbor distances,^50,64^ a systematic shift of the oscillations toward lower frequencies is observed with increasing hydration. This behavior is consistent with a progressive replacement of chloride ligands by water molecules in the first coordination shell of Co^2+^, resulting in shorter average metal–ligand distances.^71–73^ Moreover, the increase in oscillation amplitude with rising water content indicates a concurrent growth in the number of molecules coordinating the Co^2+^ photoabsorber in the first solvation shell.

However, because the experimental XAS spectra arise from the superposition of signals from coexisting cobalt species, a direct quantitative interpretation of these trends is not straightforward. To disentangle the individual structural contributions and to quantify the evolution of metal speciation as a function of hydration, the XAS data were therefore analyzed using MCR methods, as discussed in the following section.

### MCR Results

The normalized XAS spectra collected across the series exhibit well-defined and persistent isosbestic points (Figure 2a), providing strong evidence that the spectral evolution upon dilution arises from interconversion among a limited number of cobalt-containing species, rather than from a continuous distribution of coordination environments.^73^

To quantitatively determine the number and nature of the species contributing to the data set, MCR was applied to the complete set of spectra (Figure 3), with Principal Component Analysis (PCA) used to assess the effective dimensionality of the data.^44,74,75^ The Scree test performed on the PCA results shows that three principal components account for the significant spectral variance, indicating that the data set can be satisfactorily reconstructed as linear combinations of three chemically distinct cobalt species (Figure S1). During the MCR procedure, one component was constrained to ensure chemically meaningful convergence. In particular, on the basis of the UV–Vis and XAS experimental evidence, the third component (PC3) was fixed to match the reference spectrum of the Co^2+^ ion in aqueous solution, corresponding to the fully hydrated octahedral complex [Co­(H_2_O)_6_]^2+^. The remaining two components were then extracted without additional constraints. The first extracted component (PC1) closely resembles the XAS spectrum of a tetrahedrally coordinated Co^2+^species,^76^ in agreement with the UV–Vis evidence. The second component (PC2) displays intermediate spectral features and is therefore assigned to a partially chlorinated octahedral species, in which the Co^2+^ ion is coordinated by a mixed first shell of chloride anions and water molecules, following the previously described UV–vis experimental results. Further support for this assignment is provided by the analysis of the EXAFS spectra shown in Figure 3b. The EXAFS oscillations associated with PC1 differ markedly from those of the other components, displaying a higher oscillation frequency and a lower amplitude. This behavior is consistent with a tetrahedrally coordinated Co^2+^ species bound to chloride ligands, characterized by longer average metal–ligand distances and a lower coordination number. In contrast, PC2 and PC3 exhibit similar EXAFS frequencies but differ in oscillation amplitude, indicating comparable coordination structures but different metal ligands. Taken together, these observations provide further evidence for the coexistence of distinct tetrahedral and octahedral coordination complexes for Co^2+^ across the investigated series.

**Figure 3:**
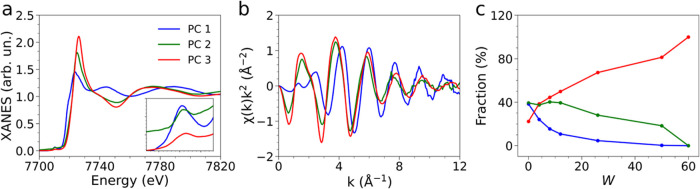
(a) XAS spectral profiles, (b) EXAFS, and (c) evolution of the relative concentration profiles with increasing molar ratio of water of MCR-derived spectral components.

The concentration profiles derived from MCR analysis (Figure 3c) describe the compositional evolution of the three cobalt species as a function of water mole fraction. In the neat MDES (W = 0), tetrahedral (PC1) and chlorinated octahedral (PC2) complexes are present in comparable amounts (40% each), while the fully hydrated species (PC3) accounts for the remaining 20%. This distribution is consistent with the UV–Vis spectrum, which displays both characteristic absorption bands, with the lower-wavelength band red-shifted relative to that of the purely hydrated complex. Upon increasing water content, PC1 markedly decreases, reflecting the progressive destabilization of chloride-rich tetrahedral complexes in increasingly aqueous environments. The chlorinated octahedral species also diminishes, albeit more gradually. In contrast, the fully hydrated [Co­(H_2_O)_6_]^2+^ complex grows steadily and becomes predominant at high water mole fractions. In the most diluted mixture (W = 50), MCR indicates approximately 80% fully hydrated species and 20% chlorinated octahedral complexes, with no detectable tetrahedral contribution, in agreement with the corresponding UV–Vis spectrum. Overall, the concentration trends obtained from MCR are fully consistent with the UV–Vis results and the systematic changes identified in the XAS spectra, providing a coherent description of cobalt speciation across the investigated composition range.

### EXAFS Results

To obtain a quantitative structural description of the species present in our system, an EXAFS analysis was performed on the spectra extracted from the MCR procedure. As previously reported in the literature,^62^ dissolution of CoCl_2_ in water at the investigated concentration (50 mM) does not lead to ion-pair formation. Instead, Co^2+^ forms a fully hydrated octahedral complex with six tightly bound water molecules in the first coordination shell.

Accordingly, the EXAFS analysis of the component associated with aqueous CoCl_2_ (PC3) was carried out by including the water Co–O and Co–H single-scattering (SS) contributions, together with the linear O–Co–O three-body MS path in the theoretical calculations. Least-squares fitting of the EXAFS spectra was performed over the *k*-range 2.1–13.9 Å^–1^ by minimizing the deviation between the experimental and theoretical curves with respect to both structural and nonstructural parameters. Following the procedure previously reported in ref 62 four parameters were refined for each SS contribution, namely the coordination number *N*, the interatomic distance *R*, the Debye–Waller factor σ^2^, and the asymmetry parameter β. In addition, two parameters, the O–Co–O bond angle and its variance, were optimized for the MS contribution. The best-fit results for the hydrated complex are shown in Figure 4 (right panel). The first three curves from the top correspond to the Co–O and Co–H SS and O–Co–O MS theoretical signals, followed by the comparison between the total theoretical signal and the PC3 experimental data, and the corresponding residuals. The agreement between the theoretical and experimental data is excellent proving the reliability of the data analysis. This conclusion is further supported by the corresponding Fourier transforms (FT) (Figure 4, lower right panel), computed over the *k*-range 2.1–13.9 Å^–1^, which display nearly identical features for the experimental and fitted curves. The optimized structural parameters are summarized in Table 1. Statistical uncertainties were evaluated from the parameter space confidence intervals following established procedures.^77,78^ For this hydrated complex, the fit yields an average of 6.0(3) oxygen neighbors at a Co–O distance of 2.09(1) Å (Table 1), consistent with previous experimental and computational studies.^63,64^ Concerning the nonstructural parameters, *E*_0_ was found to be shifted by 2.1 eV at higher values as compared to the first inflection point of the experimental spectrum, while the amplitude reduction factor *S*_0_^2^ was determined to be 0.98.

**Figure 4:**
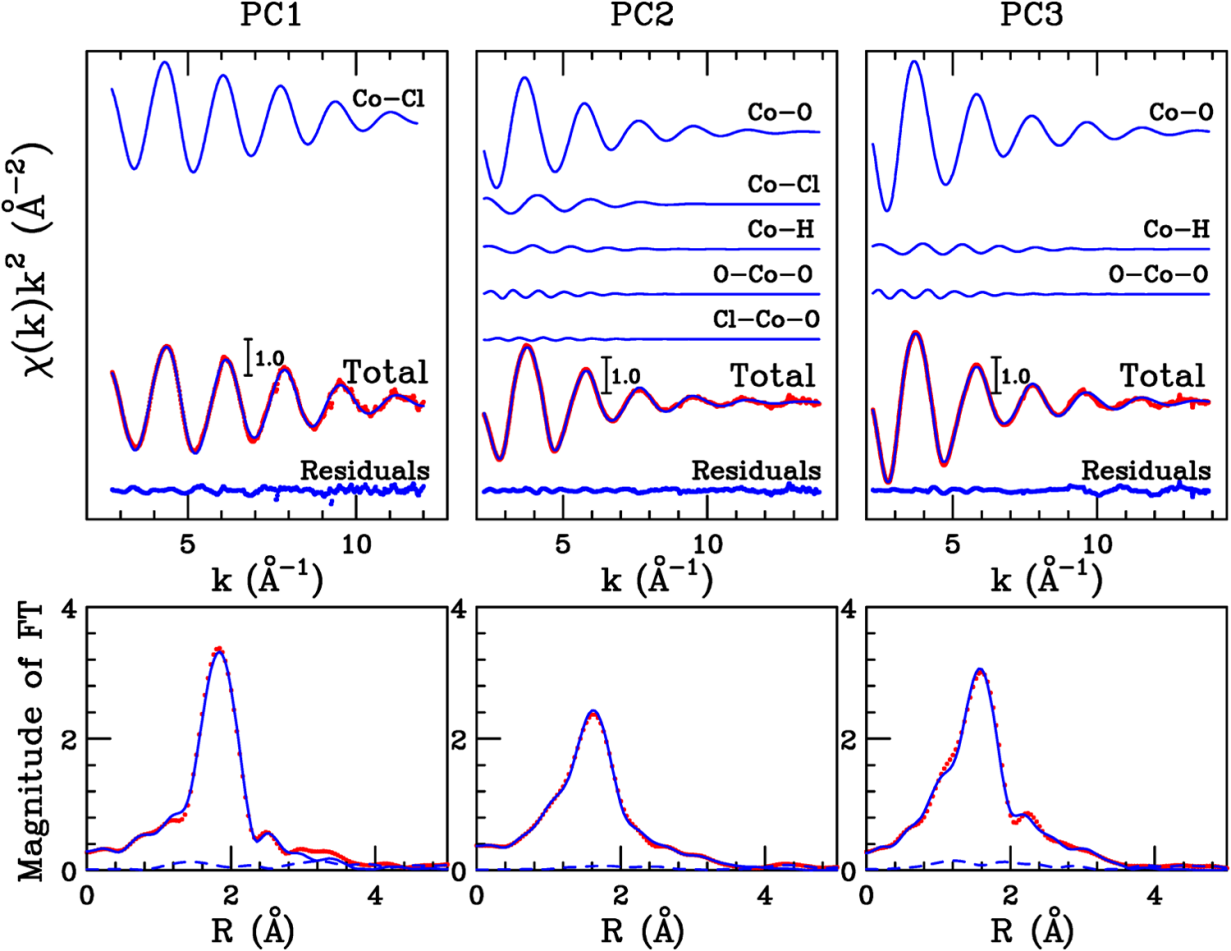
Fit of the Co K-edge EXAFS spectra of the components extracted from the MCR analysis. From top to bottom, the following curves are shown: the theoretical (a) Co–Cl, (b) Co–O, Co–Cl, Co–H, O–Co–O, and Cl–Co–O, and (c) Co–O, Co–H, and O–Co–O contributions, along with the total theoretical signal (blue line) compared with the experimental spectrum (red line). The lower panel shows the nonphase-shift-corrected Fourier Transforms (FTs) of the experimental data (red line) and the total theoretical signal (blue line) and the residual (blue dashed line).

**Table 1:** Best-Fit Structural Parameters for the Two-Body Distributions Obtained from the EXAFS Analysis of the Components Extracted from the MCR Analysis^a^

		*N*	*R* (Å)	σ^2^ (Å^–2^)	β
PC1	Co–Cl	3.9(2)	2.30(1)	0.007(2)	1.0(2)
PC2	Co–O	5.0(3)	2.09(1)	0.008(3)	0.0(3)
	Co–Cl	1.0(3)	2.34(1)	0.012(3)	0.0(3)
	Co–H	10.0(4)	2.79(2)	0.016(3)	0.1(3)
PC3	Co–O	6.0(3)	2.09(1)	0.008(3)	0.0(2)
	Co–H	12.0(4)	2.78(2)	0.012(3)	0.0(2)

a *N* is the coordination number, *R* the average distance, σ^2^ is the Debye-Waller factor, and β is the asymmetry index. Standard deviations are given in parentheses.

The EXAFS analysis of PC1 was performed, starting from a tetrahedral coordination model with four chloride ligands coordinating the Co^2+^ ion. Only the SS Co–Cl theoretical signal was included in the minimization procedure and the structural parameters associated with this signal were optimized, together with the nonstructural parameters. In this case the Cl–Co–Cl MS contribution was found to have a negligible amplitude due to the rather small angle of the three-body configuration. Least-squares refinement was carried out over the *k*-range 2.8–12.0 Å^–1^, and the resulting fits are shown in Figure 4 left panel together with the FT calculated in the same *k*-range. The agreement between the experimental and theoretical spectra, both in *k*-space and in the distance domain, is excellent. The optimized structural parameters, reported in Table 1, indicate that PC1 refers to a Co^2+^ tetrahedral complex with four chloride ions coordinating the cation at a Co–Cl distance of 2.30(1) Å. As far as the nonstructural parameters are concerned also in this case *E*_0_ was found to be shifted by 2.1 eV at higher values with respect to the first inflection point of the experimental spectrum, while the amplitude reduction factor *S*_0_^2^ was found to be 0.99.

Finally, we analyzed PC2 by starting from an octahedral coordination model. Our theoretical model included the Co–O and Co–H SS contributions from coordinated water molecules, along with the Co–Cl SS contribution from chloride ligands and the O–Co–O and Cl–Co–O MS paths. The fits were performed over the *k*-range 2.2–13.8 Å^–1^, with the constraint that the Co–O and Co–Cl coordination numbers summed to 6.0. The best-fit results are shown in Figure 4 (middle panel). The first five curves correspond to the Co–O, Co–Cl and Co–H SS theoretical signals, and the O–Co–O and Cl–O–Co three-body MS contributions; these are followed by the overlay of the total theoretical signal and the PC2 experimental data, and the residuals. The FTs of the theoretical and experimental spectra, calculated in the *k*-range 2.5–13.8 Å^–1^, are shown in the lower middle panel. In both the EXAFS and FT plots, the excellent agreement between the experimental and theoretical curves demonstrates the reliability of the results. The structural parameters are listed in Table 1. The minimization procedure indicates that PC2 corresponds to a six-coordinate complex in which the Co^2+^ ion is bound to 5.0(3) water molecules and 1.0(3) chloride ligand. The refined Co–O distance is 2.09(1) Å, consistent with previous determinations, while the Co–Cl distance is 2.34(1) Å. For this model, the fitted *E*_0_ value is 2.5 eV above the first inflection point of the experimental spectrum, and the amplitude reduction factor *S*_0_^2^ is 0.98.

### XANES Results

To further validate the reliability of the EXAFS results, we performed a quantitative analysis of the XANES region, which is particularly sensitive to the three-dimensional arrangement of atoms surrounding the photoabsorber. In particular, we carried out a fitting procedure on the XANES spectra of the components extracted through MCR with the MXAN code using the structural models identified from the combined UV–vis and EXAFS results. The XANES analysis of the PC3 spectrum has been carried out along the line of previous investigations starting from an octahedral configuration of the water molecules around the Co^2+^ cation and including the hydrogen atoms in the MXAN analysis.^64^ The best-fit results are shown in Figure 5c and the agreement between the experimental and theoretical curves is very good (*R*_sq_ = 1.6). The structural parameters obtained from the minimization procedure are reported in Table 2 and a regular octahedral Co^2+^ complex with six water ligands has been determined.^64,79,80^ Note that the Co–O distances determined by the EXAFS and MXAN analysis are equal within the reported statistical errors but there is a systematic shortening of the XANES structural determinations of about 0.03 Å as compared to the EXAFS ones. The origin of this effect has been deeply investigated^64^ and it has been found to be due to the low-energy behavior of the real part of the HL potential. The nonstructural parameters obtained from the fitting procedure are listed in Table S1.

**Figure 5:**
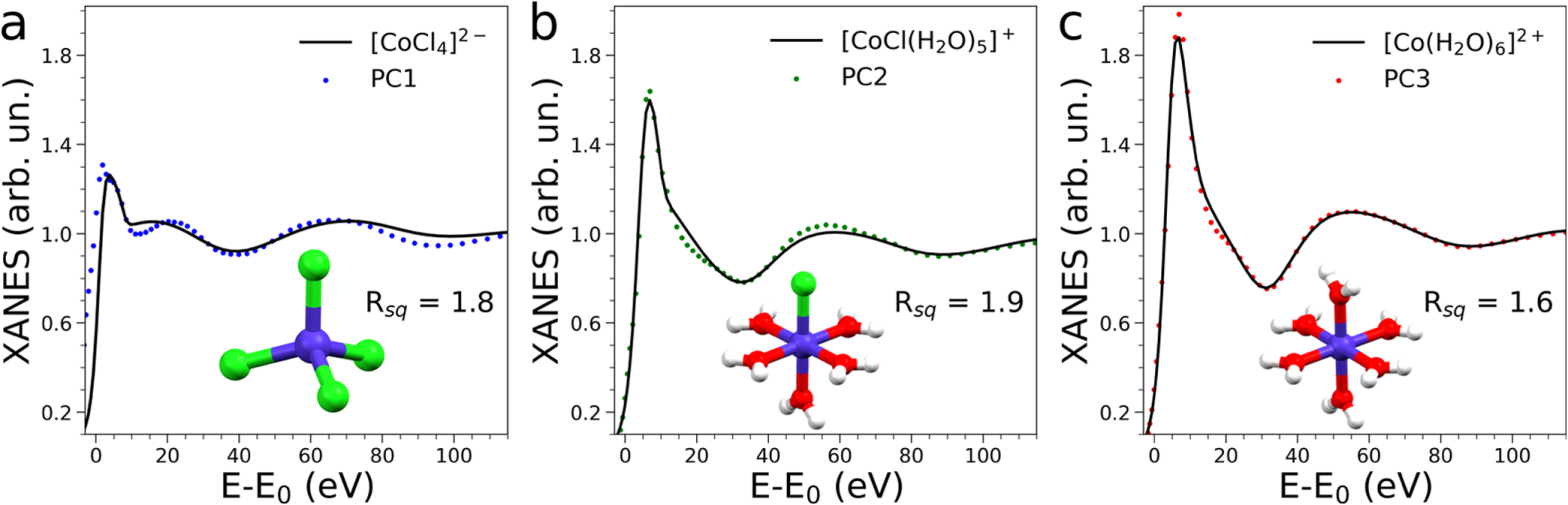
Co K-edge XANES spectra of the components extracted from the MCR analysis (dots) compared with the corresponding MXAN best-fit calculations (solid black lines). (a) Tetrahedral [CoCl_4_]^2–^ cluster; (b) mixed-ligand octahedral [CoCl­(H_2_O)_5_]^+^ cluster; (c) fully hydrated octahedral [Co­(H_2_O)_6_]^2+^ cluster. The residual functions *R*_sq_ are reported together with the optimized clusters (cobalt is purple, chlorine is green, oxygen is red, and hydrogen is white).

**Table 2:** Best-Fit Structural Parameters Obtained from the XANES Analysis of the Extracted Spectra from the MCR Analysis^a^

	*R*_Co–Cl_	*R*_Co–O_	*E*_0_	*R*_sq_
PC1	2.28(3)	-	3.0(5)	1.8
PC2	2.33(3)	2.06(3)	3.0(5)	1.9
PC3	-	2.06(3)	4.2(5)	1.6

a *R*(Å) is the average distance, *E*_0_ (eV) is the theoretical threshold energy and *R*_sq_ is the residual function.

The compatibility of the XANES spectrum of PC1 with the existence of a [CoCl_4_]^2–^ complex has been assessed by performing a minimization of the experimental data while imposing a tetrahedral geometry with four chloride ligands. In the minimization procedures the Co–Cl distances were optimized together with the nonstructural parameters. In Figure 5a we report the best-fit results where the theoretical curve corresponds to a tetrahedral model with four Cl^–^ ligands at distance of 2.28(3) Å (Table 2). Also in this case the agreement between the experimental and theoretical curve is quite good (*R*_sq_ = 1.8) and a slightly shorter Co–Cl distance has been obtained as compared to the EXAFS determination. The nonstructural parameters are similar to those obtained in the previous analysis (Table S1).

Finally, the XANES spectrum of PC2 was analyzed starting from the [CoCl­(H_2_O)_5_]^+^ model determined from the EXAFS investigation. During the minimization, five Co–O and one Co–Cl distances were optimized, while imposing an octahedral geometry and including the hydrogen atoms of the water molecules. The results of this analysis are shown in Figure 5b, and also in this case the agreement between the theoretical and experimental spectrum is very good in the whole energy range (*R*_sq_ = 1.9). The best-fit structure is a 6-fold complex with the chloride atom at 2.33(3) Å and five oxygen atoms at 2.06(3) Å (Table 2) and these results are in good agreement, within the statistical errors, with the EXAFS determinations. The nonstructural parameters are reported in Table S1, and also in this case they are quite similar to the values obtained from the previous analysis.

As a final step of our investigation, DFT calculations were performed to further validate the structural models derived from the XAS analysis. Geometry optimizations were carried out for the three cobalt complexes identified by the MCR procedure, namely [CoCl_4_]^2–^, [CoCl­(H_2_O)_5_]^+^, and [Co­(H_2_O)_6_]^2+^, both in the gas phase and using the SMD implicit solvation model. Details of the computational methodology are reported in the Supporting Information. In all cases, the optimized structures retained the coordination geometries inferred from the XAS analysis, namely tetrahedral for [CoCl_4_]^2–^ and octahedral for [CoCl­(H_2_O)_5_]^+^ and [Co­(H_2_O)_6_]^2+^ (Figure S2). The calculated structural parameters are in good agreement with those obtained from the combined EXAFS and XANES analyses (Table S2). Notably, inclusion of solvent effects systematically improves the agreement with the experimental data. For the tetrahedral [CoCl_4_]^2–^ species, the calculated Co–Cl distance decreases from 2.34 Å in the gas phase to 2.30 Å with the SMD model, matching the experimental value of 2.30(1) Å. For the mixed-ligand [CoCl­(H_2_O)_5_]^+^ complex, inclusion of solvation increases the Co–Cl distance from 2.25 Å to 2.37 Å, bringing it closer to the experimental value of 2.34(1) Å, while the Co–O distance decreases from 2.16 Å to 2.13 Å, approaching the EXAFS value of 2.09(1) Å. Finally, for the fully hydrated [Co­(H_2_O)_6_]^2+^ complex, the calculated Co–O distance of 2.12 Å is also in excellent agreement with the experimental determination of 2.09(1) Å. Overall, the DFT calculations provide independent support for the structural models obtained from the XAS analysis and confirm the importance of accounting for solvent effects when describing cobalt speciation in these highly hydrated systems.

Taken together, these results offer detailed insights into the local coordination environments within the MDES system. The excellent agreement between theoretical and experimental XANES spectra provides strong structural evidence for the species identified through MCR and EXAFS, confirming that the three components correspond to the three coordination motifs initially proposed.

## Discussion and Conclusion

The present study provides a detailed structural description of cobalt speciation in the ChCl/CoCl_2_·6H_2_O 1:2 MDES and its aqueous mixtures, demonstrating how controlled hydration modulates the coordination chemistry of the metal center. In agreement with previous UV–Vis investigations of Co^2+^ in chloride-containing environments,^52–57^ the spectroscopic results reveal the coexistence of tetrahedral and octahedral complexes in the pure MDES, with chloride-rich species favored under low-water conditions. Upon progressive water addition, the equilibrium shifts systematically toward octahedral hydration, ultimately leading to the predominance of fully hydrated [Co­(H_2_O)_6_]^2+^ complexes, consistent with earlier structural studies of aqueous cobalt solutions.^50,62–64^

The EXAFS analysis provides quantitative structural confirmation of three distinct species, namely [CoCl_4_]^2–^, [CoCl­(H_2_O)_5_]^+^, and [Co­(H_2_O)_6_]^2+^, with refined bond distances and coordination numbers in excellent agreement with literature data for tetrahedral chlorocomplexes and octahedral aquo complexes.^30,50,62–64^ The identification of the mixed-ligand [CoCl­(H_2_O)_5_]^+^ species provides direct structural evidence for stepwise ligand substitution within the MDES matrix, highlighting the intermediate role of partial hydration. These findings confirm that even moderate water contents significantly perturb the local coordination environment, with likely consequences for macroscopic properties such as viscosity, conductivity, and electrochemical performance, in line with previous studies emphasizing the strong influence of water in DES and MDES systems.^26,27^

The observed hydration-driven evolution of cobalt speciation can also be interpreted in terms of the structural reorganization of the MDES hydrogen-bond network. Previous studies on ChCl-based DESs have shown that chloride ions play a central role as hydrogen-bond acceptors, interacting with both hydrogen-bond donor species like the cholinium cation and the hydration water molecules associated with the metal salt.^81,82^ These interactions contribute to the disruption of the crystal lattices of the solid starting components and to the stabilization of the eutectic liquid through an extended supramolecular network. Upon water addition, this network undergoes a progressive reorganization, as water increasingly competes for chloride coordination and becomes the dominant hydrogen-bonding species. As a consequence, the specific chloride–choline and chloride–water interactions characteristic of the neat MDES are gradually weakened, while the cobalt coordination equilibrium shifts from chloride-rich complexes toward hydrated octahedral species. At high water contents, the predominance of the [Co­(H_2_O)_6_]^2+^ complex indicates that the local environment around the metal ion increasingly resembles that of a pure aqueous solution.

A similar hydration-driven reorganization has recently been reported for Ce^3+^ in ChCl:urea/water mixtures, where neutron diffraction measurements combined with Empirical Potential Structure Refinement simulations revealed a progressive evolution of the metal coordination environment upon water addition.^83^ In both systems, increasing water content promotes the formation of increasingly water-rich coordination environments, accompanied by changes in the metal coordination number and local geometry, driving a gradual transition from DES-like to aqueous-like environments.^83–85^ The agreement between these independent studies, despite the different metal ions and DES compositions investigated, suggests that hydration-driven restructuring of the metal solvation shell may represent a general feature of metal-containing DES/water mixtures.^83^ Nevertheless, important differences arise from both the distinct chemistry of the metal ions and the DES composition. While Ce^3+^, owing to its larger ionic radius, exhibits higher coordination numbers and a marked preference for oxygen donor ligands, Co^2+^ forms a distribution of chloride-rich and hydrated complexes whose relative abundance is strongly dependent on the water content. In this context, the combination of UV–Vis spectroscopy, XANES/EXAFS analysis, and MCR decomposition employed in the present work provides a detailed characterization of the cobalt speciation equilibria across the entire hydration range, complementing previous structural investigations of metal-containing DES/water mixtures.

A comparison with the urea:NiCl_2_·6H_2_O MDES investigated by Busato et al.^30^ further highlights the central role of water in metal-based eutectic systems. In that case, water was demonstrated to be essential for MDES formation, as the eutectic phase could not be obtained from the anhydrous salt. Molecular dynamics simulations revealed an oligomeric network of hydrated nickel chlorocomplexes linked through chloride ions and hydrogen bonds, with water acting as both hydrogen-bond donor and acceptor. In the Ni-based system, water is an integral structural component of the eutectic architecture, and in the MDES Ni^2+^ adopts a mixed coordination environment consisting on average of one chloride and five water ligands, progressively evolving toward a fully hydrated octahedral complex upon dilution without the formation of tetrahedral species. Although the specific coordination geometries differ because of the distinct electronic preferences of Ni^2+^ and Co^2+^, both systems clearly demonstrate that water governs the coordination equilibria through stepwise ligand substitution and hydrogen-bond network reorganization, thereby controlling the structural organization of the MDES.

From a methodological standpoint, this work underscores the importance of integrating complementary spectroscopic techniques with multivariate analysis. UV–Vis spectroscopy provides rapid qualitative insight into coordination geometry but lacks quantitative structural resolution. XAS offers detailed local structural information, yet the spectra represent averaged contributions in multicomponent systems. The application of MCR enables the deconvolution of overlapping spectral features, allowing individual species to be isolated and structurally characterized. In addition, both EXAFS and XANES refinements benefit from qualitative UV–Vis data used as an initial input to guide the analysis, thereby establishing a self-consistent methodological loop. This strategy is consistent with recent advances in multivariate-assisted XAS analysis for complex liquid environments^44,74,75^ and proves particularly effective for elucidating metal speciation in MDES matrices.

Overall, the combined UV–Vis/XAS/MCR approach delivers a coherent and internally consistent description of cobalt coordination across the entire hydration range. Beyond the specific ChCl/CoCl_2_·6H_2_O system, the methodology developed here represents a general and transferable strategy for investigating metal speciation in DESs and related complex fluids. Considering the growing technological relevance of MDESs in electrodeposition, catalysis, and energy-related applications, a detailed understanding of hydration-dependent coordination equilibria is essential for rational solvent design. The present study therefore contributes both fundamental structural insight and a robust protocol applicable to a broad class of metal-containing eutectic systems.

## Supplementary Material



## Notes

Additional details on the multivariate analysis, DFT calculations, and structural characterization of cobalt species (PDF)
