# Supplementary material for: A Combined Spectroscopic and Multivariate Analysis Approach for the Structural Characterization of Metal-Based Deep Eutectic Solvents: Choline Chloride and Cobalt Chloride Hexahydrate in Water Mixtures
Source: Inorg Chem. 2026 Jul 14;65(29):16738–48. doi: 10.1021/acs.inorgchem.6c01344 (PMC13418167; doi:10.1021/acs.inorgchem.6c01344)
Supplement: Supplementary file 1 [file ic6c01344_si_001.pdf]

Supporting Information: A Combined Spectroscopic and Multivariate Analysis Approach for the Structural Characterization of Metal-Based Deep Eutectic Solvents: Choline Chloride and Cobalt Chloride Hexahydrate in Water Mixtures

Giorgia Mannucci[a], Alessandro Tofoni[a], Matteo Busato[a], Mauro Giustini[a], and Paola D'Angelo[a]*

[a]*Department of Chemistry, Sapienza University of Rome, P.le A. Moro 5, 00185 Rome, Italy*

E-mail: p.dangelo@uniroma1.it

Contents

1 Determination of the number of principal components through the scree plot statistical test S-3

2 Density Functional Theory calculations S-3

3 Supplementary Figures S1- S2 S-5

4 Supplementary Tables S1- S2 **S-6**

References **S-7**

1 Determination of the number of principal components through the scree plot statistical test

We applied a scree plot analysis to the Co K-edge XAS spectra to assess the number of pure species present in solution.

It can be shown that

$$\lambda_i = \frac{\sigma_{ii}^2}{m-1} \tag{1}$$

where σ_{ii} are the singular values obtained from the Singular Value Decomposition (SVD) procedure and λ_i are the eigenvalues of the covariance matrix of $\mathbf{D}$ (evaluated over m energy points) associated with the i-th component. These values correspond to the variance explained by each principal component (PC). Consequently, components characterized by large σ_{ii} values contribute significantly to the reconstruction of the dataset, whereas components with small σ_{ii} values are primarily associated with noise. In a scree plot, the singular values associated with each principal component are plotted as a function of the component number. The presence of an elbow in the resulting curve enables the separation of signal-related components from those associated with noise. Figure S1 shows that the first three principal components capture the significant spectral variance, suggesting that the dataset can be satisfactorily reconstructed using three chemically distinct cobalt species.

2 Density Functional Theory calculations

Density functional theory (DFT) optimizations were carried out with the ORCA quantum chemistry program, version 6.1.1.[S1] Calculations were performed using the ωB97X-D3BJ functional, while convergence was enforced using the KDIIS and second-order self-consistent field (SOSCF) solvers.[S2–S6] To account for the diffuse negative charge of the Cl^- ion, the ma-def2-TZVP basis set was used for Cl while all other atoms were treated with the def2-TZVP basis.[S7] The resolution

of identity/chain of spheres exchange (RIJCOSX) approximation, making use of an automatically constructed auxiliary basis set (AutoAux setting),[S8,S9] was also employed to reduce the computational cost associated with the fine grid (DEFGRID3 setting) chosen to achieve high accuracy in the numerical DFT integration. The $[Co(H_2O)_6]^{2+}$, $[CoCl(H_2O)_5]^{+}$, and $[CoCl_4]^{2-}$ clusters were optimized both in the gas phase and using the SMD solvation model to account implicitly for solvent effects. Water was chosen as the solvent because specific SMD parameters for MDESs are not currently available.[S10] The optimized structures are shown in Figure S2. Inclusion of the solvent model resulted in minor rearrangements of the cluster geometry, mainly consisting in the reorientation of hydrogen atoms, together with a significant shift of the bond distances toward the experimental values determined in the EXAFS and XANES analyses. The average Co–O and Co-Cl bond lengths and O–Co–O, O-Co-Cl and Cl-Co-Cl bond angles obtained from the DFT optimization of the $[Co(H_2O)_6]^{2+}$, $[CoCl(H_2O)_5]^{+}$, and $[CoCl_4]^{2-}$ clusters in the gas phase and with the SMD model are reported in Table S2. Analytical frequency calculations were performed for all optimized structures to verify the absence of imaginary vibrational frequencies, confirming that each structure corresponds to a true minimum on the potential energy surface.

3 Supplementary Figures S1- S2

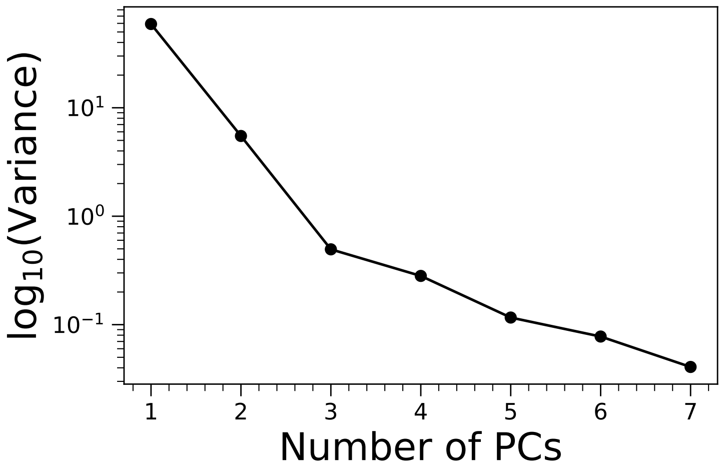

Figure S1: Scree plot statistical analysis of the Co K-edge XAS data shown in Figure 2a.

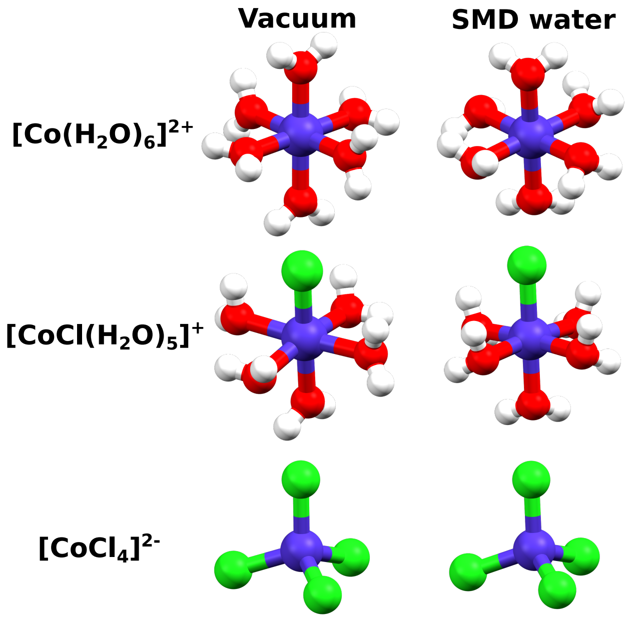

Figure S2: $[Co(H_2O)_6]^{2+}$, $[CoCl(H_2O)_5]^{+}$, and $[CoCl_4]^{2-}$ clusters optimized through DFT in vacuum and SMD H_2O. Atom color code: cobalt, purple; chlorine, green; oxygen, red; hydrogen, white.

4 Supplementary Tables S1- S2

Table S1: Best-fit nonstructural parameters obtained from the XANES analysis of the extracted spectra from the MCR analysis. E_F (eV) is the energy of the Fermi level, Γ_c (eV) is the the core-hole life-time, Γ_{exp} (eV) is the experimental resolution, E_s (eV) and A_s are the energy and amplitude of the plasmon, respectively

	E_F	Γ_c	Γ_{exp}	E_s	A_s
PC1	-1.7	1.1	1.5	8.2	22.0
PC2	-3.0	1.8	1.5	8.1	14.0
PC3	-2.5	1.6	1.3	12.0	8.5

Table S2: Average Co–X bond lengths and X–Co–X bond angles (X = O or Cl) obtained from the DFT optimization of the $[Co(H_2O)_6]^{2+}$, $[CoCl(H_2O)_5]^{+}$, and $[CoCl_4]^{2-}$ clusters in the gas phase and with the SMD model.

Cluster	Model	d_{Co-O} (Å)	d_{Co-Cl} (Å)	O–Co–O (°)	O–Co–Cl (°)	Cl–Co–Cl (°)
$[CoCl_4]^{2-}$	Gas phase	–	2.34	–	–	109.5
	SMD	–	2.30	–	–	109.4
$[CoCl(H_2O)_5]^{+}$	Gas phase	2.16	2.25	172.3	179.0	–
	SMD	2.13	2.37	174.9	179.4	–
$[Co(H_2O)_6]^{2+}$	Gas phase	2.12	–	180.0	–	–
	SMD	2.12	–	178.7	–	–

References

(S1) Neese, F. Software Update: The ORCA Program System, Version 6.0. *WIREs Comput. Mol. Sci.* **2025**, *15*, e70019.

(S2) Chai, J.-D.; Head-Gordon, M. Systematic Optimization of Long-Range Corrected Hybrid Density Functionals. *J. Chem. Phys.* **2008**, *128*, 084106.

(S3) Neese, F. Approximate Second-Order SCF Convergence for Spin Unrestricted Wavefunctions. *Chem. Phys. Lett.* **2000**, *325*, 93–98.

(S4) Grimme, S.; Ehrlich, S.; Goerigk, L. Effect of the Damping Function in Dispersion Corrected Density Functional Theory. *J. Comput. Chem.* **2011**, *32*, 1456–1465.

(S5) Bykov, D.; Petrenko, T.; Izsak, R.; Kossmann, S.; Becker, U.; Valeev, E.; Neese, F. Efficient Implementation of the Analytic Second Derivatives of Hartree-Fock and Hybrid DFT Calculations. *Mol. Phys.* **2015**, *113*, 1961–1977.

(S6) Grimme, S.; Antony, J.; Ehrlich, S.; Krieg, H. A Consistent and Accurate Ab Initio Parametrization of Density Functional Dispersion Correction (DFT-D) for the 94 Elements H-Pu. *J. Chem. Phys.* **2010**, *132*, 154104.

(S7) Weigend, F.; Ahlrichs, R. Balanced Basis Sets of Split Valence, Triple Zeta Valence and Quadruple Zeta Valence Quality for H to Rn: Design and Assessment of Accuracy. *Phys. Chem. Chem. Phys.* **2005**, *7*, 3297–3305.

(S8) Helmich-Paris, B.; de Souza, B.; Neese, F.; Izsák, R. An Improved Chain of Spheres for Exchange Algorithm. *J. Chem. Phys.* **2021**, *155*, 104109.

(S9) Stoychev, G. L.; Auer, A. A.; Neese, F. Automatic Generation of Auxiliary Basis Sets. *J. Chem. Theory Comput.* **2017**, *13*, 554–562.

(S10) Marenich, A. V.; Cramer, C. J.; Truhlar, D. G. Universal Solvation Model Based on Solute Electron Density and on a Continuum Model of the Solvent Defined by the Bulk Dielectric Constant and Atomic Surface Tensions. *J. Phys. Chem. B* **2009**, *113*, 6378–6396.
